# Hyperspectral retinal imaging to detect Alzheimer’s disease in a memory clinic setting

**DOI:** 10.1186/s13195-025-01887-4

**Published:** 2025-10-28

**Authors:** Ana Luiza Dallora, Jan Alexander, Pushpa Priyanka Palesetti, Diego Guenot, Madeleine Selvander, Johan Sanmartin Berglund, Anders Behrens

**Affiliations:** 1 Department of Health, Blekinge Institute of Technology, Valhallavägen 10, Karlskrona, 371 79 Sweden; 2 Mantis Photonics AB, Lund, Sweden; 3 Department of Clinical Sciences Malmö, Lund University, Lund, Sweden; 4 Sundets Ögonläkare, Lomma, Sweden; 5 Department of Medicine, Blekinge Hospital, Karlskrona, Sweden

**Keywords:** Alzheimer’s disease, Cognitive impairment, Amyloid-beta (Aβ), Biomarker, Retina, Cerebrospinal fluid, Hyperspectral imaging, Memory clinic, Machine learning, Catboost

## Abstract

**Background:**

Previous literature indicate retinal hyperspectral imaging as a non-invasive method with the potential for identifying amyloid-beta (Aβ) protein deposits. Current diagnostic methods, such as cerebrospinal fluid analysis or positron emission tomography, are costly, invasive, and non-scalable. Hyperspectral imaging offers a potentially accessible alternative for early detection of Alzheimer’s disease. The aim of this study is to investigate the potential of retinal hyperspectral imaging in identifying Aβ-positive patients within a clinical cohort from a memory clinic.

**Methods:**

A prospective cross-sectional cohort study was conducted between January 2023 and May 2024 at a single memory clinic in Sweden. The study recruited 57 patients (35 Aβ-positive and 22 Aβ-negative) who underwent lumbar puncture as part of their diagnostic workup for cognitive complaints. Retinal hyperspectral images were captured from all participants at the time of their lumbar puncture and again 2–4 weeks later. Data was collected from five anatomical regions of the retina (Superior 1, Superior 2, Inferior 1, Inferior 2, and the center of the Fovea).The main outcome was the Aβ status (Aβ-positive or Aβ-negative). Catboost machine learning models were trained on hyperspectral imaging data to predict Aβ status. A nested cross-validation approach was used to train and evaluate classification models. Performance metrics included area under the curve (AUC), accuracy, sensitivity, and specificity.

**Results:**

The best-performing model used the combination of regions Superior 1, Superior 2, and center of the fovea, achieving a mean AUC of 0.77 (0.05), mean accuracy of 0.66 (0.03), and mean sensitivity of 0.73 (0.13) and mean specificity of 0.55 (0.12). Performance was consistent across outer folds. Models using all five regions or less-informative combinations yielded lower and more variable results.

**Conclusions:**

Retinal hyperspectral imaging combined with the Catboost algorithm demonstrated significant potential as a non-invasive biomarker for detecting Alzheimer’s disease in a consecutive clinical cohort. Further studies should validate these findings in larger, more diverse populations and explore the integration of hyperspectral imaging with other diagnostic modalities. Limited sample size and imaging constraints highlight the need for validation in diverse clinical settings.

**Trial registration:**

ClinicalTrials.gov, ID: NCT05604183 (registration date: 2022-10-27).

**Supplementary Information:**

The online version contains supplementary material available at 10.1186/s13195-025-01887-4.

## Background

The definition of Alzheimer’s Disease (AD) is shifting from a syndromic to a biological definition [1]. This has been driven by the recognition of the syndromic overlap with other cognitive disorders and the evidence of variability in disease expression, with a long preclinical phase, followed by a phase with minimal symptoms, making a purely clinical/syndromic diagnosis challenging [2, 3]. Studies comparing the diagnosis of AD with clinical/syndromic criteria to that of autopsy show a high misdiagnosis rate, especially regarding the specificity performance measure (44–71%) [4], and the rate of misdiagnosis might be even higher in primary care where the most patients are managed [5]. The shift has also been driven by emerging therapies targeting core pathology in the disease, making an etiological diagnosis vital for successful treatment [6]. In this pursuit, there has been a focus on biomarkers for in-vivo assessing the pathological state.

The hallmark proteins of AD are Amyloid Beta (Aβ), which forms insoluble fibrillar Aβ aggregates of extracellular plaques about 20 years before symptom onset, and tau, which becomes hyperphosphorylated and forms intracellular neurofibrillary tangles that drive neurodegeneration and cognitive decline [7]. Aβ accumulation can reliably be assessed through Ab-PET or cerebrospinal fluid (CSF) analysis [8], but cost, availability and invasiveness limit the scalability of these methods. Efforts to improve the diagnosis and management of dementia focus on enhancing the accuracy of clinical diagnoses through advanced neuroimaging and blood biomarkers [2, 3, 9].

Delayed or missed diagnoses of dementia in primary care remain a significant issue, driven by factors such as patient-provider communication and system resource constraints [4]. The development of novel antibody therapies targeting Aβ represents a breakthrough in AD treatment, offering hope for disease modification and improved clinical outcomes [5]. Longitudinal studies tracking Aβ deposition, cerebral atrophy, and cognitive decline provide valuable insights into the progression of AD from its preclinical phase to fully expressed clinical syndrome, highlighting the importance of early intervention and continuous monitoring [6].

An emerging area of research focuses on retinal analysis using hyperspectral imaging (HSI), which shows promising potential for diagnosing AD. The retina exhibits many of the same cellular, structural, and molecular characteristics as the brain, including the presence of neurons, glial cells, and a blood–tissue barrier [10, 11]. Further, studies have shown a correlation between Aβ levels in the retina and in the brain, with other neuropathological assessments [12, 13]. HSI is a technology that captures information in a wide band of the electromagnetic spectrum and allows for identification of elements based on their unique spectral signatures. HSI and optical coherence tomography are promising non-invasive techniques for detecting retinal changes, providing spectral information that helps distinguish between AD patients and controls [11, 14, 15]. The main hypothesis is that protein accumulations cause frequency-dependent shifts in the reflected light spectrum that can be captured by hyperspectral images.

Typical limitations of hyperspectral retinal cameras are the low spatial and spectral resolution of the camera (measuring the light in a limited number of wavebands and pixels), and slow image acquisition, making images prone to movement artifacts i.e., eye movements [14]. New developments in this technology employ a broader spectral range and higher resolution, addressing aforementioned limitations, and making this technology suited for retinal imaging and applications in ophthalmology and neurology [16].

AD detection with hyperspectral retinal images, combined with machine learning techniques, have shown promising results in proof-of-concept studies in human subjects [11, 13, 15]. However, the performance of the method has not been yet assessed in a clinical setting, more specifically in a memory clinic setting where all of the patients have cognitive complaints. By detecting spectral shifts caused by retinal protein accumulations, such as beta-amyloid, and, combined with advanced data analysis and machine learning, HSI could offer a non-invasive, faster, and more cost-effective method for AD detection compared to traditional techniques.

This study aims to evaluate the performance of HSI in detecting Aβ-positive individuals within a clinical cohort from a memory center.

## Methods

### Study design

This study was performed as a single-center prospective cross-sectional cohort study, recruiting consecutive patients referred for a lumbar puncture as part of their diagnostic workup at a memory clinic in Karlskrona, Sweden (ClinicalTrials.gov, ID: NCT05604183, registration date: 2022-10-27). Data collected from retinal HSI was used to build binary classification models with the CSF beta-amyloid 42/40 ratio as outcome measure.

### Recruitment

Individuals investigating cognitive complaints at the memory clinic are referred for a lumbar puncture as part of their diagnostic workup. The participants were recruited for the study when they presented for this procedure at the Neurology Clinic of the Blekinge Hospital in Karlskrona, Sweden. At the time of their first visit, the patients were informed about the research. Upon obtaining informed consent, participants underwent retinal scanning in addition to the lumbar puncture, which was performed as a standard part of clinical care, independent of the decision to participate in the research. The participants were invited back 2–4 weeks after the procedure for an additional retinal scan and a computerized cognitive testing.

The inclusion criteria for participation were: (i) consent to participate in the study; (ii) no contraindications for mydriatic drops; (iii) performed lumbar puncture procedure of standard care and (iv) at least 18 years old. Recruitment was carried out between January 2023 and May 2024, during which all eligible patients were invited to participate.

Sample size determination was based on an effect size approach, following the methodology described by Salgado (2018) [17]. Previous studies using hyperspectral retinal imaging for amyloid status classification reported diagnostic performance corresponding to an area under the curve (AUC) of approximately 0.74 [11, 13]. This AUC corresponds to a Cohen’s d of 0.909 and an odds ratio of 5.19. Assuming a balanced distribution of Aβ-positive and Aβ-negative cases, a type I error rate of 5%, and a power of 80%, the minimum required number of events per predictor was calculated to be approximately 12 per group.

Given the effect size and the need to support model training and validation, a conservative total sample size of at least 50 subjects was defined. Recruitment was planned with the goal of including at least 30 amyloid-positive and 30 amyloid-negative participants. This sample size was expected to leave around 24 participants per group for training the model after holding out cases for validation, which is slightly higher than the typically recommended by standard methodological guidelines.

### Ethical considerations

The study was approved by the Ethical Review Board in Gothenburg (Dnr 2022–05804-01) and was conducted in accordance with the Declaration of Helsinki. Written informed consent was obtained from all participants. All acquired data were anonymized and stored under the security protocols of the Blekinge Institute of Technology.

### General evaluation

All patients had an evaluation at the memory clinic. Cognitive staging (Subjective cognitive impairment, Mild cognitive impairment, Dementia) was performed by a medical doctor specialized in cognitive medicine. Cognitive screening was performed with the Montreal Cognitive Assessment (MoCA) test [18]. Cognitive symptoms were assessed with the Cognitive Impairment Questionnaire (CIMP-QUEST) [19], which records topographic symptoms in MCI and dementia. Symptoms of depression were assessed with the Geriatric Depression Scale (GDS) [20] from the computerized cognitive test Computer Cognitive Testing in Patients With Mild Cognitive Impairment (CoGNIT) [21], performed on a tablet computer on the second visit at the neurology clinic. The data from this general evaluation, along with sociodemographic data, including age, gender, and education was collected for sample descriptive statistics. The Additional File 1 provides detailed information on the diagnostic procedures of the study’s setting.

### Cerebrospinal fluid analysis

The spinal tap was performed by a neurologist (last author) in the L3-L4 or L4-L5 interspace, with the patient in the sitting position, using a 22-gauge Quincke spinal needle. Information about sample handling is provided on the Additional File 1.

The outcome measure of the experiment was the CSF beta-amyloid 42/40 ratio, determined through laboratory analysis of material collected via lumbar puncture. Participants with the FDA approved cutoff below 0.72 were classified as amyloid positive cases (Aβ-positive), otherwise amyloid negative cases (Aβ-negative). This is sufficient to diagnose AD adhering to the 2024 revised diagnostic criteria from the Alzheimer Association Work group [1].

### Hyperspectral imaging of the retina

In this study, a snapshot hyperspectral camera was used, requiring only a single acquisition. Each capture was performed in a single flash (≈ 6 ms), minimizing motion artifacts related to eye movements.

The input information for building the classification models was obtained through HSI of the retina. HSI is the capability to capture image data across a wide range of wavelengths simultaneously, providing detailed spectral information for each pixel in a scene [22].

Mydriatic eye-drops (Topicamide 0.5% and Phenylephrine 10%) were applied approximately 20 min before the procedure. Both retinae were photographed with the snapshot hyperspectral camera CB200-10 nm-160 × 160-2 developed by Mantis Photonics AB and described by Guenot et al. (2024) [16] (Swedish Medical Products Agency registry: CIV-22-06-039726), connected with a C-mount to a TL-230T relay lens mounted on a Topcon TRC-50IX fundus camera. It provides images of a 50 × 50 degree field-of-view of the retina with 150 × 150 pixels and 18 nm spectral resolution in the 450–700 nm spectral band. The spectrum was sampled at 7 nm, generating 36 wavelength channels. The images were obtained by either a trained medical doctor or by a trained technician in the presence of a medical doctor.

**Fig. 1:**
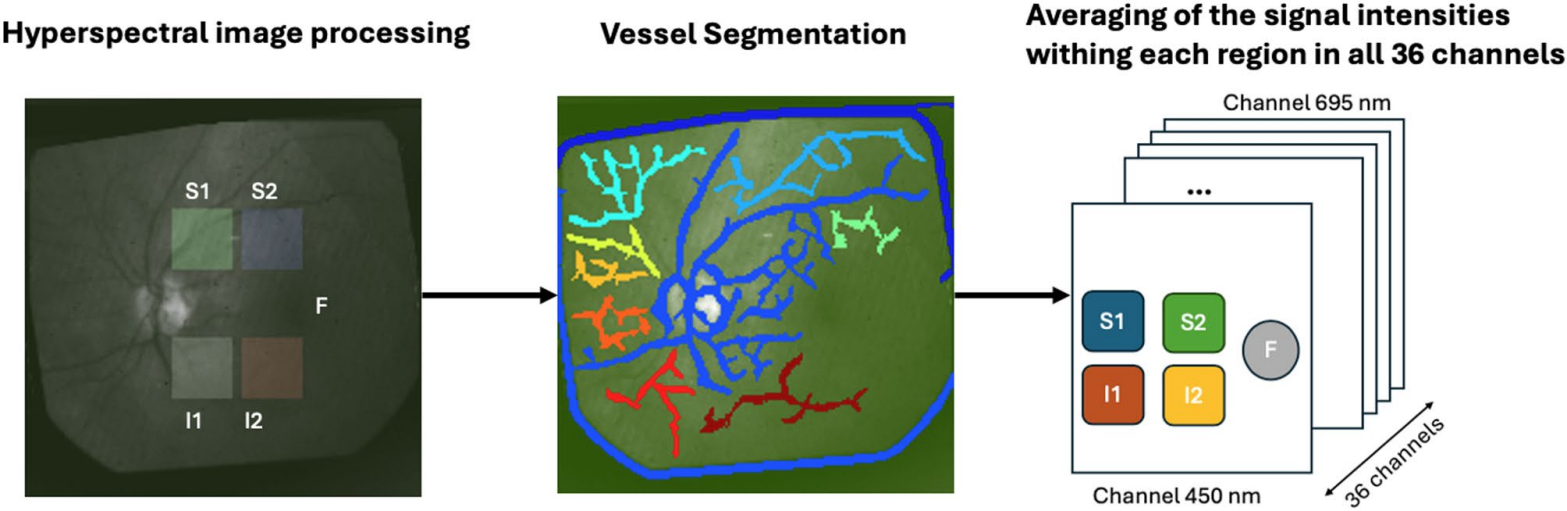
Overview of the data preprocessing procedure

Five anatomical landmark regions of the retina were annotated in each image (see Fig. 1). The fovea was sampled using a circular area of interest (20-pixel diameter) in the center of the fovea (F). Areas in the temporal vascular arcades, superior (S1) and inferior (I1) to the fovea, as well as superior (S2) and inferior (I2) to the ONH, were sampled using squares (25 × 25 pixels) on template horizontally oriented with the temporal raphe based on the annotated reference points.

**Fig. 2:**
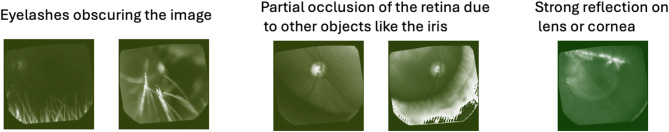
Reasons that led to the exclusion of images for the analysis

The annotation was carried out by a technician who was blinded to the CSF analysis results. Both retinae were scanned, but the highest quality image from each session was selected for annotation. The images were evaluated for flaws, excluding those with obstructions like eyelashes, the iris, or light reflections, as well as out-of-focus images (see Fig. 2). Only clear, well-focused images showing the optic disc, macula, and vessel arc were chosen.

Systematic sampling locations were then semi-automatically selected to ensure reproducibility and prevent bias. Specialized software from the HSI camera was used to manually mark the center of the fovea and optic nerve head. The orientation of the temporal raphe was calculated, and five sampling locations from key retinal structures were automatically extracted for each image.

Vessel segmentation was employed to remove vessel pixels that could interfere with the analysis of amyloid protein aggregates in the modeling phase. Given the considerable variability in retinal vascular anatomy between individuals, each sampling location might contain differing numbers of pixels overlying blood vessels. Retinal blood vessels have a prominent spectral signature [23], and failing to exclude them could introduce unnecessary variability in the spectral profile. To address this, blood vessels visible in the inner retina were automatically segmented using the 555 nm slice of the hypercube image. This segmentation was performed by Gaussian filtering for image smoothing, then ridge detection based on the Hessian derivative matrix. The process included normalization for contrast adjustment, colormap application for better visualization, and the creation of a binary mask to represent the segmented vessels [24].

Finally, the average intensity for each region was calculated in each of the 36 spectral channels (ranging from 450 nm to 695 nm, in 7 nm intervals).

### Binary classification experiment

Binary classification models were built with input data of the average intensity of each region (S1, S2, I1, I2 and F) for each of the 36 spectral channels (450 nm to 695 nm), and main outcome measure of the Aβ status (Aβ-positive or Aβ-negative) given by the CSF analysis. CatBoost [25] and Principal Component Analysis were employed to address the high dimensionality and the redundant correlated data given by the hyperspectral images. These techniques have demonstrated efficacy in similar contexts [26–28].

A nested cross-validation (nCV) framework was used to evaluate model performance and tune hyperparameters while reducing the risk of overfitting. Both inner and outer loops used grouped, stratified 3-fold cross-validation to maintain class balance and ensure that no data from the same individual appeared in both training and test sets. Within each inner loop, a randomized search over 50 hyperparameter configurations was performed. Parameter ranges included: tree depth (4–10), learning rate (0.01–0.3), number of iterations (100–400), L2 regularization (1–10), border count (50–250), bagging temperature (0–1), and random strength (1–10). Performance was evaluated using the average area under the curve (AUC) across inner folds, and the best-performing configuration was used to retrain the model on the full outer training set. This approach mitigates information leakage and supports an unbiased evaluation of generalization performance on the outer test sets.

Experiments were repeated across all combinations of one, two, three, four, and five retinal regions, resulting in a total of 31 region-based experiments. Performance metrics (AUC, accuracy, sensitivity, specificity) were given for each of the outer test sets. No formal sensitivity analysis was conducted. However, model robustness was assessed by evaluating a wide range of input combinations, allowing us to examine how different anatomical configurations influenced performance. No missing data handling was necessary, as flawed or low-quality images were excluded prior to analysis, and the final dataset was complete.

All analyses were conducted in RStudio (v2024.04.1 + 748), using the following R packages: catboost for model training and prediction, pROC for AUC computation, rsample and caret for cross-validation, and dplyr, tidyr, and purrr for data processing.

### Statistics

Difference between Aβ-positive and Aβ-negative participants were explored with the t-test for continuous variables and the chi-square tests for proportions. The significance level for all analyses was set to 0.05. Statistical analyses were performed in SPSS (Version 28; IBM Corp, Armonk, NY).

## Results

### Sample characteristics

Sixty-two patients were initially eligible for the study. 1 patient declined participation, 1 was excluded due to an unsuccessful spinal tap, and 3 were excluded due to insufficient retinal image quality. Feature extraction was performed on images from 57 of the 60 patients, selecting the highest quality image from each session. Images were excluded based on the following reasons: (i) interference from external elements such as hair or eyelashes; (ii) centrally positioned optical discs that did not capture the full vessel arc and macula; (iii) inadequate illumination; and (iv) misalignment leading to partial imaging of the iris rather than the retina. The final analysis included 88 images from 57 patients.

Table 1 presents the demographic, clinical characteristics and diagnostic findings of the participants. 35 participants were Aβ-positive and 22 Aβ-negative based on the results of the CSF analysis. There were no significant differences in age or gender. Differences were observed in educational backgrounds with the Aβ-positive participants more often having university-level education compared to Aβ-negative participants, who more frequently had only primary or high school education. Aβ-positive participants had significantly lower MoCA scores [18] and lower GDS scores [29], indicating greater cognitive impairment and more severe depressive symptoms. CSF biomarkers, including Amyloid 42/40 ratio, pTau, and tTau levels, were significantly different between the groups, reflecting the pathological changes associated with AD, while CSF-Nfl levels did not differ significantly.

**Table 1:** Demographics, clinical data, and diagnostic findings (*N* = 57 participants) in terms of the means (standard deviations), and the statistical significance given by the t-tests or chi-square tests

	Aβ-positive (*n* = 35)	Aβ-negative (*n* = 22)	*p*-value
Age (years)	69.7 (5.0)	69.2 (5.1)	0.7
Gender (female %)	20 (57%)	11 (50%)	0.6
Education (primary/highschool/university)	9/8/18	7/11/4	< 0.05
Symptom duration (years)	1.5 (0.7)	1.7 (1.0)	0.9
Cognitive stage (Subjective cognitive impairment/Mild cognitive impairment/Dementia)	0/33/2	2/19/1	0.2
MoCA (points)	22.9 (3.4)	24.9 (3.5)	< 0.05
GDS (points)	2.7 (2.2)	5.0 (4.9)	< 0.05
Memory symptoms measured by the CIMP-QUEST (points)	5.8 (3.6)	6.1 (3.8)	0.72
CSF-Amyloid 42/40	0.47 (0.11)	0.99 (0.11)	< 0.001
CSF-pTau	83.8 (44.2)	33.2 (11.9)	< 0.001
CSF-tTau	551 (285)	338 (199)	< 0.01
CSF-Nfl	1755 (1402)	1633 (1775)	0.78

### Experimental results

For the binary classification experiments, the number of principal components was fixed at five, to balance dimensionality reduction with variance retention. Given the high number of hyperspectral input features, using too few components risks underfitting by discarding potentially informative variation. Additionally, due to the limited sample size, the nCV setting was set to three inner and three outer folds to ensure sufficient training data within each fold.

Table 2 presents the performance metrics for the best-performing combination of anatomical regions at each region count, with results reported for each outer fold (see Additional File 2 for the results for all combinations of regions). The best overall performance was achieved with the three-region combination S1, S2, and F, showing consistent results across all folds with AUCs ranging from 0.71 to 0.80. Sensitivity remained relatively high (0.65–0.88), while specificity also improved compared to other configurations.

**Table 2:** Classification performance metrics across outer folds for the best-performing region combinations. Each row represents a single outer fold result for the specified region set

Regions	*n*CV outer fold	AUC	Accuracy	Sensitivity	Specificity
S2	1	0.64	0.55	0.47	0.67
S2	2	0.70	0.50	0.45	0.60
S2	3	0.48	0.62	0.67	0.55
S1, S2	1	0.63	0.59	0.71	0.42
S1, S2	2	0.81	0.73	0.80	0.60
S1, S2	3	0.65	0.55	0.56	0.55
S1, S2, F	1	0.80	0.69	0.88	0.42
S1, S2, F	2	0.71	0.63	0.65	0.60
S1, S2, F	3	0.79	0.66	0.67	0.64
S1, S2, I1, F	1	0.76	0.66	0.76	0.50
S1, S2, I1, F	2	0.65	0.53	0.45	0.70
S1, S2, I1, F	3	0.74	0.66	0.72	0.55
All	1	0.58	0.52	0.65	0.33
All	2	0.53	0.60	0.75	0.30
All	3	0.62	0.55	0.67	0.36

Including additional regions beyond S1, S2, and F did not improve performance, suggesting added noise due to redundant input features. The four-region model (S1, S2, I1, F) achieved comparable but slightly more variable results, and the model using all five regions demonstrated diminished performance.

Table 3 summarizes the mean performance metrics across folds for each of the best combinations of regions. The S1, S2, F combination not only achieved the highest mean AUC (0.77) but also demonstrated the low variability across folds with a standard deviation of 0.05 even in the small sample setting.

**Table 3:** Mean classification performance for each region combination across outer folds, presented as mean (standard deviation (SD))

Regions	Mean AUC (SD)	Mean Accuracy (SD)	Mean Sensitivity (SD)	Mean Specificity (SD)
S2	0.61 (0.11)	0.56 (0.06)	0.53 (0.12)	0.6 (0.06)
S1, S2	0.7 (0.1)	0.62 (0.1)	0.69 (0.12)	0.52 (0.09)
S1, S2, F	0.77 (0.05)	0.66 (0.03)	0.73 (0.13)	0.55 (0.12)
S1, S2, I1, F	0.71 (0.06)	0.61 (0.07)	0.65 (0.17)	0.58 (0.1)
All	0.57 (0.05)	0.56 (0.04)	0.69 (0.05)	0.33 (0.03)

The best performing combinations consistently involved the same anatomical regions, namely S1, S2, and F. This pattern was observed across multiple configurations, including the two, three, and four region models. These results suggest that superior retinal regions (S1 and S2) and the fovea (F) may carry particularly informative spectral features for distinguishing amyloid-positive from amyloid-negative individuals. The stability of these regions across the highest-performing models strengthens their potential as target zones for future imaging and diagnostic efforts.

## Discussion

### Discussion of the results

The findings of this study demonstrate the potential of HSI as a non-invasive method for detecting Alzheimer’s disease (AD) through retinal imaging in a consecutive clinical cohort. Our results indicate that retinal hyperspectral data from the superior regions (S1, S2) and the fovea (F) provided the most informative patterns for distinguishing Aβ-positive from Aβ-negative individuals as determined by CSF biomarkers. The CatBoost model, trained on HSI data from these regions, achieved consistently good classification performance, with the three-region combination S1, S2, and F yielding the highest AUC, accuracy, and stability across cross-validation folds. These findings suggest that this region combination may contain particularly informative spectral features for distinguishing Aβ status. The reduced performance observed when all five regions were included suggests that not all retinal areas contribute equally, and that including non-informative or redundant regions may introduce noise or reduce specificity.

Compared with prior research on hyperspectral retinal imaging for AD detection, the present study demonstrates competitive diagnostic performance in a real-world clinical setting. For instance, Lemmens et al. (2020) [11] reported an AUC of 0.74 using a cohort of 17 cases and 22 controls, while Hadoux et al. (2019) [13] achieved an AUC of 0.87 in a small validation set (4 cases and 13 controls). In contrast, the present study used a consecutive clinical cohort comprising individuals referred for diagnostic evaluation due to already present cognitive complaints, reflecting a real-world memory clinic population, which is not the case for the aforementioned studies. As shown in Table 1, the groups did not differ significantly in age, gender, cognitive stage classification or memory symptoms, but did show variation in MoCA scores, education levels, and CSF biomarkers, showing the heterogeneity typically encountered in clinical practice.

Despite this difference, the best-performing region combination (S1, S2, and F) in our study achieved an average AUC of 0.77, with consistent performance across folds. This result may point to the potential of hyperspectral imaging to detect Alzheimer’s disease pathology highlighting its diagnostic relevance.

While earlier studies emphasized the diagnostic potential of the S2 region, our findings extend this by identifying S1, S2, and F as the most informative anatomical combination.The fact that these areas are most relevant is also consistent with previous reports of amyloid beta deposits in post mortem flat mounted retinas, preferentially affecting the superior retina [30]. The superior and foveal regions provided informative spectral features associated with Aβ status, supporting their relevance as targets in future HSI-based detection strategies. Further research is warranted to explore multimodal approaches, such as combining HSI with optical coherence tomography (OCT), which Lemmens et al. [14] found to marginally improve performance.

The diagnostic performance from HSI alone seems lower than the best performing blood-based biomarker [31, 32] for AD detection, particularly the plasma biomarker pTau217, which has shown accuracy up to 90% in diagnosing AD in both primary and secondary care settings [31]. Though not directly compared, HSI may have some advantages. Blood biomarkers are a more indirect measure that could be influenced by renal function and the integrity of the blood-brain barrier function potentially affecting accuracy in certain patient populations [33, 34]. Additionally, spurious “spikes” in pTau levels that can lead to misdiagnosis when relying on single readings [35]. A multimodal approach might be a way forward, where convergence of results from several modalities e.g. blood and HSI might indicate highly probable cases and select for subjects needing invasive confirmatory testing, a topic for further research.

In terms of practical considerations, HSI may hold an advantage over blood-based biomarkers with respect to cost per scan. Image acquisition is performed in a single flash and does not require consumables. The current post-processing pipeline requires approximately 20 min, primarily for extracting hyperspectral data from the raw sensor output. However, with GPU acceleration this processing time can be reduced to only a few seconds, making real-time analysis feasible. By contrast, blood-based biomarkers involve laboratory assays, specialized equipment, and higher recurring costs, which may limit scalability in some healthcare settings.

It can be argued that HSI may be more comparable to amyloid-PET, as it measures cumulative effects rather than production/clearance rates like biofluid analysis, making it more resilient to biological fluctuations. Evidence suggests that amyloid beta (via the Aβ 42/40 ratio) is an earlier biomarker than pTau217 in AD progression [36], offering potential for earlier or even presymptomatic diagnosis. HSI could also be widely accessible, given the common use of fundus cameras in ophthalmology and optometry, without the need for specialized labs or equipment for mass spectrometry or immunoassays.

The limited specificity observed in our models may in part be explained by overlapping retinal changes associated with other types of dementia. Studies report the pathological accumulation of α-synuclein in the retina for Lewy body dementia and Parkinson’s disease [37–39]. Vascular cognitive impairment is associated with vascular alterations that may also affect retinal reflectance properties [40]. These pathologies could contribute to false-positive classifications in hyperspectral imaging. Future studies with more diverse cohorts should investigate how such comorbidities and differential diagnoses specifically influence hyperspectral signatures in the retina.

### Limitations

Several limitations should be noted. The small sample size may limit the generalizability, especially outside specialized memory clinics. While a more accessible biomarker could see broader use in primary care, future validation studies in such settings are needed for HSI, as seen with AD blood biomarkers. The imaging process poses challenges, as hyperspectral imaging is sensitive to eye movement and poor illumination, potentially affecting accuracy. Technological improvements, such as adding autofocus, could mitigate these issues. Although multiple testing is a concern, consistent model metrics across various combinations suggest this risk is minimized.

An additional limitation is the variability in classification performance across folds. The best-performing model (S1, S2, F) achieved AUCs ranging from 0.71 to 0.80. These values indicate reasonable discrimination, but the range also highlights that the model cannot yet be considered robust as a biomarker. It is important to note that the present experiments were conducted in a memory clinic setting where all participants had cognitive complaints. These findings show potential for further development of HSI.

A methodological limitation is that the HSI approach in this study provides a dichotomized classification of Aβ status, whereas established biomarkers such as PET and CSF yield continuous measures that can support disease staging, particularly for tau pathology. Plasma biomarkers are emerging as promising continuous measures, but they also show substantial intra-individual variability due to both biological and analytical factors, which reduces their reliability for fine-grained staging [41]. Further developments may enable HSI to produce more nuanced outputs.

Finally, an important consideration is the potential influence of common ocular diseases on HSI performance, which was outside the scope of the present study. Glaucoma causes thinning of the retinal nerve fiber layer and structural changes in the optic nerve head, which increase relative reflectance at longer wavelengths in HSI [42]. Age-related macular degeneration has been shown to exhibit distinct spectral signals detectable by HSI and shares pathological mechanisms such as oxidative stress and extracellular deposits with Alzheimer’s disease [43]. Cataract increases light scattering in the ocular media, reducing retinal image contrast and producing a broad spectral attenuation across wavelengths more marked at shorter wavelengths compared to longer wavelengths [44, 45]. While this study did not assess ocular comorbidities, future studies should systematically investigate how prevalent eye diseases may influence hyperspectral features relevant to Alzheimer’s disease.

## Conclusion

This study highlights the potential of HSI as a non-invasive tool for detecting AD. The CatBoost model demonstrated promising classification performance, particularly when using spectral data from the superior retinal regions (S1, S2) and the fovea (F), which showed the strongest association with amyloid-β status confirmed via cerebrospinal fluid biomarkers. While these findings are encouraging, further research is needed in larger, more diverse populations, especially in earlier clinical stages and primary care settings. Future work may also explore the integration of HSI with other accessible biomarkers and cognitive assessments to improve diagnostic precision. Additionally, the broader applicability of HSI in distinguishing other neurodegenerative conditions, such as Lewy body and vascular dementia, warrants investigation.

## Supplementary Information

Additional file 1.

Additional file 2.

## Data Availability

The datasets used and/or analysed during the current study are available from the corresponding author on reasonable request.

## Abbreviations

Aβ: Amyloid–beta
AD: Alzheimer’s Disease
AUC: Area under the Curve
CIMP-QUEST: Cognitive Impairment Questionnaire
CoGNIT: Computer Cognitive Testing in Patients With Mild Cognitive Impairment
CSF: Cerebrospinal fluid
F: Center of the fovea
GDS: Geriatric Depression Scale
HSI: Hyperspectral imaging
I1: Inferior region to the fovea (temporal vascular arcades)
I2: Inferior region to the fovea
MoCA: Montreal Cognitive Assessment
OCT: Optical coherence tomography
S1: Superior region to the fovea (temporal vascular arcades)
S2: Superior region to the fovea
SD: Standard deviation
