# Supplementary material for: Hyperspectral retinal imaging to detect Alzheimer’s disease in a memory clinic setting
Source: Alzheimers Res Ther. 2025 Oct 28;17:232. doi: 10.1186/s13195-025-01887-4 (PMC12570430; doi:10.1186/s13195-025-01887-4)
Supplement: Supplementary file 1 — Additional file 1. [file 13195_2025_1887_MOESM1_ESM.pdf]

Additional File 1

This document provides detailed information on the diagnostic procedures used to evaluate patients in the study, including the general clinical assessment, stepwise dementia investigation process in the study setting, and the methods for cerebrospinal fluid collection and analysis of Alzheimer's disease biomarkers.

Patients' general evaluation

The investigation of a patient in Blekinge county, where the study was carried out, is performed according to regional guidelines[1]. The investigation of cognitive impairment is carried out in two steps: a basic dementia investigation and an extended dementia investigation. The basic investigation is performed in primary care by a general practitioner and a dementia nurse, including an interview with the patient and their family, cognitive screening tests, (i.e., Swedish revision of the Mini-Mental State Examination (MMSE-SR)[2], Clock drawing Test[3], Cube test[4], Rowland Universal Dementia Assessment Scale (RUDAS)[5], Montreal Cognitive Assessment (MOCA)[6], and quick test of cognitive speed[7]), assessment of Activities of Daily Living[8], medical examination, blood tests, electrocardiogram , body mass index, orthostatic blood pressure test, and computed tomography of the brain. Depending on the result of the basic investigation, the patient may be referred to the memory unit for an extended investigation. The following patients are referred to the memory unit in Karlskrona for an extended dementia investigation after the basic dementia investigation has been completed:

- Patients with suspected dementia who are relatively young, usually under 65 years of age.
- Patients with very early signs of memory impairment, i.e. mild cognitive impairment.
- Patients with difficult-to-diagnose or atypical conditions with memory impairment, personality changes, etc.
- Patients with rapid dementia progression.
- Patients where a second opinion is desired.

Before the referral is sent from primary care to the memory unit, the basic investigation must be completed. The basic investigation includes computed tomography, blood sampling, electrocardiogram and cognitive screening tests by a dementia nurse, such as MMSE-SR/RUDAS and Clock Test. The extended dementia investigation is carried out at the memory unit in Karlskrona and includes a more in-depth assessment of the patient's cognitive functions, including a cognitive screening with the MoCA-test and a neuropsychological examination. Other investigations that may be performed include magnetic resonance imaging, regional cerebral blood flow (rCBF), electroencephalogram, and cerebrospinal fluid analysis (CSF).

Cerebrospinal fluid analysis

The spinal tap was performed by a neurologist (last author) in the L3-L4 or L4-L5 interspace, with the patient in the sitting position, using a 22-gauge Quincke spinal needle. According to clinical routine at the neurology department, CSF was collected in 4 tubes where the third tube was used for routine CSF analyses and the fourth sample was used for AD biomarkers (Ab42, Ab42/Ab40-ratio, pTau18, tTau and neurofilaments). CSF was collected in polypropylene tubes and gently mixed to avoid gradient effects. All samples were centrifuged within 30 minutes at +4uC at 2000 g for 10 min to remove cells and debris. To analyze the Alzheimer disease (AD) markers, samples were centrifuged before transport and 0.5 mL was frozen (-80°C) in cryotubes and sent frozen to the Sahlgrenska University Hospital clinical chemistry laboratory. The core AD biomarkers were analyzed by a fully automated immunoassay instrument LUMIPULSE G (Fujirebio)[9]. The FDA approved limit of an AB42/40-ratio below 0.72 was used for classification of amyloid positive cases. Neurofilament light chain was analyzed with an ELISA method[10].

References

1. Blekinge R. Vårdprogram demens. Region Blekinge. August 13, 2024. Accessed October 28, 2024. https://regionblekinge.se/halsa-och-vard/for-vardgivare/vardprogram-demens.html
2. Palmqvist S. MMSE-SR: the Standardized Swedish MMSE (2nd version). Svensk Förening för Kognitiva sjukdomar. Published online 2013.
3. Watson YI, Arfken CL, Birge SJ. Clock completion: an objective screening test for dementia. J Am Geriatr Soc. 1993;41(11):1235-1240. doi:10.1111/j.1532-5415.1993.tb07308.x
4. Maeshima S, Osawa A, Maeshima E, et al. Usefulness of a cube-copying test in outpatients with dementia. Brain Inj. 2004;18(9):889-898. doi:10.1080/02699050410001671847
5. Storey JE, Rowland JTJ, Basic D, Conforti DA, Dickson HG. The Rowland Universal Dementia Assessment Scale (RUDAS): a multicultural cognitive assessment scale. Int Psychogeriatr. 2004;16(1):13-31. doi:10.1017/s1041610204000043
6. Nasreddine ZS, Phillips NA, Bédirian V, et al. The Montreal Cognitive Assessment, MoCA: a brief screening tool for mild cognitive impairment. J Am Geriatr Soc. 2005;53(4):695-699. doi:10.1111/j.1532-5415.2005.53221.x
7. Kvitting AS, Wimo A, Johansson MM, Marcusson J. A quick test of cognitive speed (AQT): usefulness in dementia evaluations in primary care. Scand J Prim Health Care. 2013;31(1):13-19. doi:10.3109/02813432.2012.751699
8. Lawton, Brody. Instrumental activities of daily living scale (IADLS) and physical self-maintenance scale (PSMS). In: A Compendium of Tests, Scales and Questionnaires. Psychology Press; 2020:452-456. doi:10.4324/9781003076391-130
9. Gobom J, Parnetti L, Rosa-Neto P, et al. Validation of the LUMIPULSE automated immunoassay for the measurement of core AD biomarkers in cerebrospinal fluid. Clin Chem Lab Med. 2022;60(2):207-219. doi:10.1515/cclm-2021-0651

10. Gaetani L, Höglund K, Parnetti L, et al. A new enzyme-linked immunosorbent assay for neurofilament light in cerebrospinal fluid: analytical validation and clinical evaluation. Alzheimers Res Ther. 2018;10(1):8. doi:10.1186/s13195-018-0339-1
