# Supplementary material for: Hyperspectral retinal imaging to detect Alzheimer’s disease in a memory clinic setting
Source: Alzheimers Res Ther. 2025 Oct 28;17:232. doi: 10.1186/s13195-025-01887-4 (PMC12570430; doi:10.1186/s13195-025-01887-4)
Supplement: Supplementary file 2 — Additional file 2. [file 13195_2025_1887_MOESM2_ESM.pdf]

Additional file 2

The Additional Table 1 shows the results regarding all combinations of regions in the experiments: Superior 1 (S1), Superior 2 (S2), Inferior 1(I1), Inferior 2 (I2) and Center region of the Fovea (F).

Additional Table 1. Performance metric results of the 31 experiments with combinations of regions S1, S2, I1, I2 and F

Regions	Outer fold number	AUC	Accuracy	Sensitivity	Specificity
S1	1	0.61	0.55	0.71	0.33
S1	2	0.50	0.57	0.65	0.40
S1	3	0.74	0.72	0.72	0.73
S2	1	0.64	0.55	0.47	0.67
S2	2	0.70	0.50	0.45	0.60
S2	3	0.48	0.62	0.67	0.55
I1	1	0.57	0.62	0.59	0.67
I1	2	0.62	0.53	0.45	0.70
I1	3	0.61	0.62	0.72	0.45
I2	1	0.54	0.66	0.71	0.58
I2	2	0.50	0.43	0.45	0.40
I2	3	0.51	0.59	0.72	0.36
F	1	0.54	0.66	0.71	0.58
F	2	0.50	0.43	0.45	0.40
F	3	0.51	0.59	0.72	0.36
S1, S2	1	0.63	0.59	0.71	0.42
S1, S2	2	0.81	0.73	0.80	0.60
S1, S2	3	0.65	0.55	0.56	0.55
S1, I1	1	0.42	0.55	0.65	0.42
S1, I1	2	0.57	0.50	0.45	0.60
S1, I1	3	0.72	0.66	0.67	0.64
S1, I2	1	0.52	0.59	0.71	0.42
S1, I2	2	0.52	0.50	0.50	0.50
S1, I2	3	0.72	0.66	0.78	0.45
S1, F	1	0.47	0.45	0.53	0.33
S1, F	2	0.51	0.47	0.45	0.50
S1, F	3	0.68	0.62	0.72	0.45
S2, I1	1	0.59	0.52	0.71	0.25
S2, I1	2	0.59	0.50	0.50	0.50
S2, I1	3	0.76	0.69	0.72	0.64

S2, I2	1	0.68	0.62	0.59	0.67
S2, I2	2	0.57	0.47	0.40	0.60
S2, I2	3	0.56	0.48	0.61	0.27
S2, F	1	0.62	0.62	0.76	0.42
S2, F	2	0.59	0.60	0.65	0.50
S2, F	3	0.83	0.79	0.78	0.82
I1, I2	1	0.63	0.66	0.71	0.58
I1, I2	2	0.70	0.53	0.35	0.90
I1, I2	3	0.75	0.69	0.72	0.64
I1, F	1	0.55	0.52	0.71	0.25
I1, F	2	0.56	0.50	0.45	0.60
I1, F	3	0.47	0.55	0.72	0.27
I2, F	1	0.56	0.59	0.65	0.50
I2, F	2	0.48	0.60	0.70	0.40
I2, F	3	0.47	0.59	0.72	0.36
S1, S2, I1	1	0.55	0.59	0.65	0.50
S1, S2, I1	2	0.52	0.53	0.50	0.60
S1, S2, I1	3	0.59	0.55	0.72	0.27
S1, S2, I2	1	0.61	0.62	0.71	0.50
S1, S2, I2	2	0.53	0.57	0.65	0.40
S1, S2, I2	3	0.54	0.59	0.67	0.45
S1, S2, F	1	0.80	0.69	0.88	0.42
S1, S2, F	2	0.71	0.63	0.65	0.60
S1, S2, F	3	0.79	0.66	0.67	0.64
S1, I1, I2	1	0.58	0.59	0.65	0.50
S1, I1, I2	2	0.49	0.60	0.65	0.50
S1, I1, I2	3	0.62	0.62	0.78	0.36
S1, I1, F	1	0.47	0.55	0.65	0.42
S1, I1, F	2	0.53	0.50	0.55	0.40
S1, I1, F	3	0.74	0.76	0.89	0.55
S1, I2, F	1	0.42	0.48	0.47	0.50
S1, I2, F	2	0.58	0.57	0.65	0.40
S1, I2, F	3	0.68	0.66	0.78	0.45
S2, I1, I2	1	0.67	0.45	0.59	0.25
S2, I1, I2	2	0.58	0.60	0.55	0.70
S2, I1, I2	3	0.57	0.66	0.67	0.64
S2, I1, F	1	0.57	0.66	0.88	0.33
S2, I1, F	2	0.57	0.43	0.40	0.50
S2, I1, F	3	0.70	0.59	0.67	0.45

S2, I2, F	1	0.61	0.52	0.53	0.50
S2, I2, F	2	0.58	0.47	0.60	0.20
S2, I2, F	3	0.75	0.69	0.78	0.55
I1, I2, F	1	0.63	0.59	0.71	0.42
I1, I2, F	2	0.58	0.63	0.80	0.30
I1, I2, F	3	0.68	0.66	0.78	0.45
S1, S2, I1, I2	1	0.59	0.62	0.65	0.58
S1, S2, I1, I2	2	0.68	0.67	0.65	0.70
S1, S2, I1, I2	3	0.75	0.69	0.78	0.55
S1, S2, I1, F	1	0.76	0.66	0.76	0.50
S1, S2, I1, F	2	0.65	0.53	0.45	0.70
S1, S2, I1, F	3	0.74	0.66	0.72	0.55
S1, S2, I2, F	1	0.67	0.59	0.59	0.58
S1, S2, I2, F	2	0.66	0.70	0.75	0.60
S1, S2, I2, F	3	0.50	0.59	0.78	0.27
S1, I1, I2, F	1	0.66	0.66	0.71	0.58
S1, I1, I2, F	2	0.54	0.50	0.40	0.70
S1, I1, I2, F	3	0.73	0.69	0.78	0.55
S2, I1, I2, F	1	0.61	0.55	0.65	0.42
S2, I1, I2, F	2	0.68	0.47	0.35	0.70
S2, I1, I2, F	3	0.64	0.72	0.83	0.55
S1, S2, I1, I2, F	1	0.58	0.52	0.65	0.33
S1, S2, I1, I2, F	2	0.53	0.60	0.75	0.30
S1, S2, I1, I2, F	3	0.62	0.55	0.67	0.36
